# Prevalence and associated factors influencing the use of antibiotics for self-medication among Chinese residents: a cross-sectional study in 2021

**DOI:** 10.1186/s13690-025-01579-3

**Published:** 2025-04-15

**Authors:** Danni Wang, Pu Ge, Dong-mei Xue, Chen Hu, Guo Huang, Jiale Qi, Wen-ying Hong, Lutong Pan, Xiao Han, Jinzi Zhang, Ayidana Kaierdebieke, Rong Ling, Wen-li Yu, Ying Bian, Yibo Wu

**Affiliations:** 1 State Key Laboratory of Quality Research in Chinese Medicines, University of Macau, Macau, China; 2 Institute of Chinese Medical Sciences, University of Macau, Macau, China; 3 Faculty of Health Sciences, Department of Public Health and Medicinal Administration, University of Macau, Macau, China; 4 School of Traditional Chinese Medicine, Beijing University of Chinese Medicine, Beijing, China; 5 Nanjing University of Science and Technology ZiJin College, Nanjing, China; 6 School of Pharmaceutical Sciences, Peking University, Beijing, China; 7 International Research Center for Medicinal Administration, Peking University, Beijing, China; 8 School of Journalism&Communication, Zhengzhou University, Zhengzhou, China; 9 Faculty of Health Sciences, University of Macau, Macau, China; 10 School of public health, Shandong University, Jinan, China; 11 The Fifth Affiliated Hospital of Sun Yat-sat University, Zhuhai, China; 12 School of Humanities and Social Sciences, Harbin Medical University, Harbin, China; 13 School of public health, Xi’an Jiaotong University, Xi’an, China; 14 School of Pharmaceutical Sciences, Jilin University, Jilin University, Changchun, China; 15 School for Sports Humanities and Social Science, Jilin Sport University, Changchun, China; 16 School of Public Health, Peking University, Beijing, China

**Keywords:** Self-medication_1_, Antibiotics_2_, Family health_3_, Depression_4_

## Abstract

**Background:**

Self-medication with antibiotics (SMA) is increasingly occurring worldwide, yet it is a process that can lead to inappropriate use of antibiotics, with potentially multiple adverse consequences such as an increased risk of antibiotic resistance.

**Objective:**

The objective of this study is to assess the prevalence of self-medication with antibiotics among the Chinese population and investigate the factors associated with this behavior.

**Methods:**

A multi-stage sampling method was employed to carry out a national cross-sectional questionnaire survey among Chinese individuals aged 18 years and above from July 10, 2021, to September 15, 2021. Following the statistical analysis of the collected data, binary logistic regression was applied to identify the factors associated with respondents’ self-medication with antibiotics. Model robustness testing was also performed using best subset regression.

**Results:**

From a total of 11,031 questionnaires, 9,344 qualified samples were selected. The prevalence of self-medication with antibiotics among Chinese residents was found to be 34.63% (3,237/9,344). The most important factor considered in SMA was the advice of medical professionals, accounting for 89.00%, including recommendations from doctors (2,524/3,237, 77.97%) and pharmacists (1,905/3,237, 58.85%). The results of the binary stepwise logistic regression analysis showed that female, people older than 36 years, with higher education, had consumed alcohol in the past month, with mild depression, having residential or employee health insurance as the primary form of health coverage, having commercial insurance, having better family health status, and perceiving higher levels of social support were more likely to practice SMA (*P* < 0.05). Conversely, individuals diagnosed with major depressive disorder were found to be less likely to engage in self-medication with antibiotics (*P* < 0.05). The best subset regression method and stepwise regression method gave the same results.

**Conclusion:**

The SMA issue of Chinese residents is still relatively serious. Residents’ SMA was associated with their demographic and sociological characteristics, depression, family health, and perceived level of social support. The primary consideration for resident SMA is the advice of medical staff. The problem of SMA in China should be improved through antibiotic management, education on antibiotic knowledge, and medical staff’s correct guidance.

**Supplementary Information:**

The online version contains supplementary material available at 10.1186/s13690-025-01579-3.

**Table:** 

**Text box 1. Contributions to the literature**
• There is limited evidence on self-medication with antibiotics (SMA) in developing countries, and the prevalence of self-medication with antibiotics (SMA) among Chinese residents remains a significant public health issue
• Demographic and sociological factors, such as education, health insurance, and mental health status, are significantly associated with SMA among Chinese individuals.
• The findings indicate an urgent need for tailored antibiotic management policies and targeted educational initiatives to enhance public awareness on appropriate antibiotic use.
• This research contributes to the broader discourse on antibiotic resistance, advocating for comprehensive policies that focus on education and regulation within healthcare systems.

## Background

Antibiotic misuse may lead to significant challenges for global public health. One of the prominent manifestations of inappropriate antibiotic use is self-medication with antibiotics (SMA), which refers to the utilization of antibiotics by individuals to treat symptoms or diseases without professional advice or prescription [1]. Similar to other forms of antibiotic misuse, such as improper dosing or active alterations, SMA increases the risk of antimicrobial resistance (AMR) and can lead to serious consequences including adverse drug reactions, escalated healthcare costs, poor treatment outcomes, and heightened sequelae for patients [2, 3]. Individuals may engage in self-administration of antibiotics with an excessively broad or narrow spectrum, incorrect dosage or frequency, untimely treatment of critically ill patients, unnecessary use of antibiotics for non-indicated conditions, and procurement of antibiotics from various sources. These inappropriate practices can lead to bacterial resistance and other antibiotic-related risks. Bacteria acquire resistance to antibiotics by producing mutations. The biochemical resistance mechanisms employed by bacteria include antibiotic inactivation, target modification, altered permeability, and bypass of metabolic pathways [4, 5].

Antibiotic self-administration is widespread worldwide. A review of 36 papers published between 2013 and 2020 examined the prevalence of short-term antibiotic self-administration in Asian countries, revealing a 50.8% prevalence among residents [6]. The prevalence of SMA also varies between countries and is generally higher in developing countries [7], For example, a study conducted in 2019 among students at the National Defence University in Malaysia reported a prevalence of 39.3% for SMA [8]; another study conducted in 2014 at six different universities in Karachi, Pakistan [9], showed 47.6% of students reported SMA and another article mentioned that Pakistani pharmacists often distribute antibiotics without a prescription adding to AMR rates [10]. A 2017 study in Saudi Arabia showed approximately 34% of respondents used antibiotics without a prescription [11]. In comparison, a survey conducted in 2017 among the general population in northeastern France found a prevalence rate of only 18% for SMA. A survey conducted in 2018 on antibiotic use in the Korean population reported a prevalence rate of less than 30% for SMA. Additionally, an Australian survey found that 19.5% of 2,217 Australian adults took antibiotics to combat COVID-19 [12, 13, 14].

The incidence of SMA is also relatively high in China, where there is a significant problem of inappropriate antibiotic use despite several policies adopted by the Chinese government to promote appropriate usage. Many of these policy measures have primarily focused on restricting antibiotic prescriptions by healthcare providers and regulating the sale of antibiotics in retail pharmacies. For instance, a policy implemented in China since 2004 mandates that all retail pharmacies require a prescription for dispensing and selling drugs [15]. According to this policy, patients are not permitted to purchase antibiotics without a doctor’s prescription. However, previous studies have shown that retail pharmacies in China continue to dispense antibiotics to patients without a prescription and that the proportion of pharmacies selling antibiotics without a prescription has been reported to be high, ranging from 63–86% [16, 17]. Another survey in China found that 130 (88.4%) community pharmacies in Shenyang, Liaoning Province, in northeast China, provided antibiotics without a prescription [18, 19]. The availability of over-the-counter antibiotic medications in these retail pharmacies contributes to the risk of self-purchase and use, and self-storage of antibiotics by Chinese individuals, who have long been engaged in self-medication with antibiotics in China.

Previous studies have explored various risk factors that influence SMA, including age, education level, antibiotic knowledge, and economic circumstances. However, the profile of risk factors varies by study due to population and regional differences. Moreover, there is a limited number of surveys that have included large samples representing the entire population [20, 21, 22]. In 2018, the Chinese government released the “Pilot Work Plan for the Construction of a National Social Psychological Service System” in response to the World Health Organization’s call, placing greater emphasis on the mental health issues of Chinese residents [23]. The government also launched the “Healthy China 2030 Planning” to promote healthier behaviors among the population [24]. Both mental health and health behaviors can influence individual decision-making [25]. Against this backdrop, research examining the correlations between factors such as the mental health and health behaviors of Chinese residents and their decisions to engage in antibiotic self-medication is still insufficient. Therefore, we have included mental health issues such as depression, as well as unhealthy behaviors like alcohol consumption and smoking, as hypothesized factors in our study, aiming to expand the research possibilities related to antibiotic self-medication behaviors. Additionally, studies have indicated that health behaviors significantly impact chronic non-communicable diseases, especially in the wake of the COVID-19 pandemic [26]. We also seek to explore whether the COVID-19 pandemic has affected the antibiotic self-medication behaviors of Chinese residents; thus, we specifically chose to conduct a cross-sectional study in 2021, shortly after the outbreak of COVID-19 in 2020. Therefore, the primary objectives of this study were to assess the prevalence of SMA among Chinese individuals aged 18 years and above in 2021, understand important considerations in the purchase and use of antibiotics in the Chinese population and to identify factors associated with SMA behavior in the Chinese population.

## Materials and methods

### Study design

The data utilized in this research was obtained from the China Family Health Index-2021survey [27]. Since 2022, the survey has been renamed the Psychology and Behavior Investigation of Chinese Residents (PBICR) and studied annually [28], and the general database has been continuously updated until 2024 [25]. The data used in our current study, referred to as SHIACF-2021, involved a large sample size and was conducted nationwide between July 10th, 2021, and September 15th, 2021. After a nationwide multi-stage sampling, surveyors in each city used China’s largest online survey platform (Questionnaire https://www.wjx.cn/ [Available in Chinese only]) to administer face-to-face questionnaires to participants within their respective cities in public places. Using the SHIACF-2021 as an example, the first stage involves equal probability sampling, where four municipalities (Beijing, Tianjin, Shanghai, and Chongqing) and provincial capital cities of all provinces and autonomous regions of China are directly included in the study. For the 22 provinces and 5 autonomous regions, a sampling frame is used to determine the number of cities to be sampled based on each province’s or autonomous region’s population base, and non-capital cities are randomly selected using a random number table method. A total of 120 cities are sampled in the first stage. In the second stage, the population of each city was stratified by sex, age, and urban-rural distribution. The sample size (per 100 persons) for each stratum was determined based on the demographic characteristics derived from the results of the “7th National Population Census” [29]. At least one investigator was recruited for each city, and the investigators conducted a convenience sampling surveys while ensuring that the required sample size and proportional requirements were met.

### Participants

#### Minimum sample size

The minimum sample size was calculated using the following formula:

$$\:\:\varvec{n}=\left[{{\varvec{Z}}_{\varvec{\upalpha\:}/2}}^{2}\varvec{p}\varvec{q}\right]/{\varvec{\delta\:}}^{2}$$ [30]

In the formula n represents the sample size, p represents the estimated antibiotic self-medication rate, q = 1-p, α = 0.05, Zα/2 = 1.96 ≈ 2, δ is the permissible error, δ = 0.1*p. In 2014, a study of adult Chinese individuals conducted in China nationwide by the State showed that 23.9% of the 7,915 study participants reported that they self-medicate with antibiotics [18]. The sample size was calculated using the 23.9% prevalence of SMA in the Chinese population, and the minimum sample size calculated by substituting the formula was 1,293. **C**onsidering the possibility of 20% invalid questionnaires, the minimum number of questionnaires that should be distributed was 1,617.

#### Inclusion criteria

(1) Age > 18. (The concept of “nominal age” exists in China, and according to the Chinese nominal age system, a person is considered one year old on the day of their birth. If the birth occurs near the Lunar New Year, he or she is considered to be two years old when the Chinese New Year begins. After the child turns two years old, by the nominal age count, he or she is considered to be three years old. So to avoid including minors, respondents whose age was filled in as 18 years were excluded from this study.)

(2) Answered the SMA question (i.e., answer “yes” or “no” to the question of whether you have ever purchased and used antibiotics on your own).

(3) Participated in the study and filled in the informed consent form voluntarily.

(4) Participants that completed the network questionnaire investigation by themselves or with the help of investigators.

#### Exclusion criteria

(1) People with unconsciousness or mental disorders.

(2) People who are participating in other similar research projects.

(3) Medical practitioners. The purpose of this research was to investigate the self-medication behaviors of the public. Therefore, medical workers were excluded from this study.

**Fig. 1:**
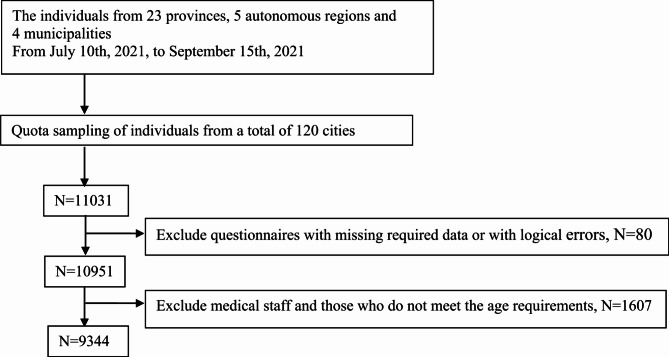
Flowchart of participant enrollment in the 2021 study in China

Therefore, we collected a total of 11,031 questionnaires, yielding 10,951 valid responses after screening, with a valid response rate of 99.27%. Following the selection criteria based on the study design, a total of 9,344 cases were included in the survey. Detailed information can be found in Fig. 1.

### Questionnaire design

The questionnaire consisted of three parts: population, sociodemographic characteristics antibiotic self-medication and scale measurements, aimed at investigating the self-medication behaviors regarding antibiotics among Chinese people and the factors influencing these behaviors. The first part focused on the socio-demographic characteristics of the population, including age, location, usual place of residence, education level, presence of cardiovascular, metabolic, and digestive diseases, presence of children, marital status, main payment method for medical expenses, current occupational status, smoking status, and alcohol consumption in the past 12 months; the second part investigated the status of individuals’ SMA behavior, as well as their considerations when purchasing antibiotics and consists of 2 questions (1 single choice question and 1 multiple choice question). The third part was composed of different types of scales, including the Perceived Social Support Scale (PSSS), the Chinese version of the short-form of the family health scale (FHS-SF Chinese version), the New General Self-Efficacy Scale (NGSES), and the Depression Screening Inventory (PHQ-9).

#### The general clinical and demographic information

The questionnaire includes a comprehensive range of information about the subjects, including their gender, age, location (eastern, central, western part of China), usual place of residence (urban, rural), education level, per capita monthly household income, marital status, primary method of medical expense coverage, current occupational status (student, employed, unemployed or retired), smoking habits, and alcohol consumption within the past twelve months.

#### Antibiotic self-medication status and important considerations when purchasing

This section consists of two questions. The first question is a single-choice question that asks participants, “Have you ever purchased and used antibiotics on your own?” Participants can choose either “yes” or “no” as their answer. The second question is a multiple-choice question designed to investigate the important considerations of participants when purchasing and using antibiotics on their own. Survey respondents who have not engaged in self-medication with antibiotics are not required to answer this question. There are 16 options provided for participants to choose from, including: (1) price of medicines; (2) efficacy of medicines; (3) safety of medicines; and (4) taste of medicines. (5) convenience of use; (6) quality of packaging; (7) dosage form; (8) brand awareness; (9) availability of reimbursement by medical insurance; (10) doctor’s advice; (11) pharmacist’s advice (Medical personnel such as nurses were not included in the questionnaire design because their knowledge of pharmacy is less comprehensive and familiar than that of clinicians and pharmacists, and nurses are usually not the primary providers of information on patient medication use); [31] (12) advice from friends and relatives; (13) personal experience; (14) advertising; (15) after-sales service; and (16) corporate reputation. For this multiple-choice question, the number of options available to the survey respondents was 1–16. For each survey respondent, the order of options for multiple-choice questions is randomized, thus reducing the generation of measurement bias.

#### The perceived social support scale (PSSS)

The Perceived Social Support Scale (PSSS) was initially developed by Blumenthal in 1987 and later translated and modified by Jiang Qianjin to form the Chinese version known as the Zimetm Perceived Social Support Scale (PSSS) [32, 33]. The PSSS is a 12-item scale divided into three subscales, including family support, friend support, and other people support. It aims to measure the level of support that individuals perceive from their family, friends, and other people. In this research Cronbach’s α for the PSSS was 0.921, indicating good reliability. The scoring method was 1 point for “strongly disagree”, 7 points for “strongly agree”, and so on. According to relevant studies, a total score between 12 and 36 is a low support status; a total score between 37 and 60 indicates an intermediate support status; and a total score between 61 and 84 indicates a high support status. Higher total scores indicate higher levels of perceived social support. For the purpose of this study, participants were divided into three groups based on their total scores: low, medium, and high subgroups [34, 35].

#### The new general self-efficacy scale (NGSES)

The New General Self-Efficacy Scale (NGSES) was adapted by Chen G et al. based on the General Self-Efficacy Scale (GSSE) to measure people’s self-efficacy. The scale consists of eight entries [36, 37]. In the current study, the Cronbach’s α coefficient for the NGSES scale was 0.943, indicating that the scale has good internal consistency. 8 items were scored on a Likert 5-point scale, with all items scored positively, from “strongly disagree” to “strongly agree”. The scores for all items were summed to obtain a total score, which represented an individual’s level of self-efficacy. A higher total score indicates better self-efficacy. In this study, the self-efficacy levels were categorized based on total scores of NGSES as follows: a total score of 0–30 was considered low self-efficacy, while a total score of 31–40 was considered high self-efficacy [38].

#### The Chinese version of the shortform of the family health scale (FHS-SF)

A Chinese version of the Family Health Scale (FHS-SF Chinese version), was translated and adapted to the Chinese context by Wang et al. [39]. The FHS-SF has 10 items divided into four dimensions, namely Family Social and Emotional Health Processes, Family Healthy Lifestyle, Family Health Resources, and Family External Social Supports. A five-point Likert scale was used, 1 meant “strongly disagree”, 5 meant “strongly agree”, and the scores for the three questions in the Family Health Resources dimension section were calculated in reverse. After reverse coding, the three items contained in the Family Health Resources section, created binary variables for each item by coding as “1” items with a score of 4 or 5 and coding as “0” those with a score less than 4. Sum of scores between 0 and 5 was considered as poor family health, a total of 6–8 points as moderate family and excellent family health as a total of 9 or10 points. The Cronbach’s α coefficient for the Family Health Questionnaire in this study was 0.848 and the Cronbach’s α coefficients for the four dimensions of Family Social and Emotional Health Processes, Family Healthy Lifestyle, Family Health Resources, and Family External Social Supports were 0.902, 0.858, 0.725, and 0.706 respectively. This indicates that the scale has good internal validity [40].

#### The depression screening inventory (PHQ-9)

The Depression Screening Inventory (PHQ-9) is the depression module of the Patient Health Questionnaire (PHQ). The PHQ-9 is one of the internationally accepted depression screening scales and is a self-rating scale that can effectively screen individuals for depression. It contains a total of 9 items, each scored on a 4-point scale (0–3). In this study, the scores were divided into four outcomes based on relevant research: scores of 0–4 indicated no depression, 5–9 suggested possible mild depression, 10–14 indicated moderate depression, and scores greater than or equal to 15 indicated severe depression [41, 42, 43]. The PHQ-9 has been found to have comparable or even higher sensitivity and specificity than other depression screening instruments and is not limited by age, gender, or ethnicity. Participants were divided into four subgroups: with no depression (0–4), mild depression (5–9), moderate depression (10–14), and major depression (greater than or equal to 15) [44].

### Statistical analysis

Statistical analyses were performed using SPSS 25.0 for Windows (SPSS, Inc., Chicago, IL, USA). The number and percentage of categorical variables were calculated. For normally distributed data, the mean and standard deviation were used for statistical description, and for non-normally distributed data, the median and interquartile range were used for statistical description. All scale scores were transformed into dichotomous variables concerning event literature, and categorical variables were presented as frequencies (percentage composition). A one-way binary logistic regression was employed to analyze the relationship between the demographic and sociological characteristics of the respondents and self-medication with antibiotics, with the independent variable for this component being the demographic and sociological characteristics of the respondents and the dependent variable the presence or absence of SMA. The independent variables consisted of the following components: age, location, usual place of residence, education level, presence or absence of cardiovascular diseases, presence or absence of metabolic diseases, presence or absence of digestive diseases, presence or absence of children, marital status, main mode the payment for medical expenses, current occupational status, whether or not smoking, whether they had consumed alcohol in the past 12 months. The rank sum test was used to test for significant differences between those who had SMA and those who had not on the scale scores of the PSSS, NGSES, FHS-SF, and PHQ-9.

A multi-factor binary stepwise logistic regression analysis was performed to examine the relationship between the dependent variable, which was whether the respondent had purchased and used antibiotics for self-medication and the independent variables including the respondent’s demographic characteristics, the scale score grading of the Family Health Scale, the PSSS scale, the new General Self-Efficacy Scale, and the Depression Screening Inventory. The optimal subset regression was performed using the bestglm package for the R language, and the model with the smallest AIC value was fitted using the AIC value as the model evaluation metric.

Various computational steps were conducted to ensure the robustness of the study results. These steps included performing common method deviation tests, best subset regression, reliability tests, and subgroup analyses.

### Quality control

The study conducted two rounds of pre-investigation and two rounds of expert consultation before the formal survey. Trained investigators distributed questionnaires to respondents and registered their codes one-on-one and face-to-face, Every Sunday evening during the investigation process, members of the research group communicated with the investigators to summarize, evaluate, and give feedback on the checks and data screening. After collecting the questionnaires, two individuals performed consecutive logic checks and data screening independently. If any outliers were identified during data analysis, the original questionnaire had to be located and verified with the investigator before proceeding to the next step of the analysis.

### Common method bias

To address the potential issue of common method bias, this research was tested by Harman’s single-factor test. The results showed that five factors exhibited eigenvalues exceeding one, and the variance contribution of the first principal factor was 34.12%, which did not exceed 40%, indicating that there was no significant common method bias [45].

## Results

### General characteristics of participants

Table 1 demonstrates the differences in demographic characteristics between groups based on whether residents used antibiotics for self-medication. A total of 9,344 subjects were included in this study, of which 3,237 (34.64%) had purchased and used antibiotics for self-medication. Of the participants, 4,342 (46.47%) were male and 5,002 (53.53%) were female. The majority of respondents fell into the age range of 19 to 35 years (46.02%) and 36 to 59 years (42.37%), more than half of the respondents lived in Eastern China, with over 70% living in urban areas and 60.24% having a higher level of education. In addition, there was a significant number of respondents with a monthly income ranging from RMB 3,001 to 6,000 ($437.11 - $873.92). It is worth noting that 75.95% of respondents primarily relied on resident/employee health insurance as their main method of paying for medical expenses.

**Table 1:** Participants’ characteristics

Variables		Number of respondents *n*(%)	Antibiotics self-medication	
			Yes	No
Self-medication with antibiotics (SMA)		3237(34.64)		
Gender				
	Male	4342(46.47)	1464(33.72)	2878(66.28)
	Female	5002(53.53)	1773(35.45)	3229(64.55)
Age				
	19–35	4300(46.02)	1367(31.80)	2933(68.20)
	36–59	3959(42.37)	1489(37.61)	2470(62.39)
	≥ 60	1085(11.61)	381(35.12)	704(64.88)
Location				
	The eastern part of China	4842(51.82)	1697(35.05)	3145(65.95)
	The central part of China	2528(27.05)	898(35.52)	1630(64.48)
	The western part of China	1974(21.13)	642(32.52)	1332(67.48)
Place of residence				
	Urban	6736(72.09)	2388(35.45)	4348(64.55)
	Rural	2608(27.91)	849(32.55)	1759(67.45)
Education level				
	Primary Education or no education	989(10.58)	307(31.04)	682(68.96)
	Secondary education	2727(29.18)	968(35.50)	1759(64.50)
	Tertiary education	5628(60.24)	1962(34.86)	3666(65.14)
Monthly income (RMB)				
	≤ 3000	2755(29.48)	921(33.43)	1834(66.57)
	3001–6000	3656(39.13)	1283(35.09)	2373(64.91)
	≥ 6000	2933(31.39)	1033(35.22)	1900(64.12)
Marital status				
	Not married	3118(33.37)	999(32.04)	2119(67.96)
	Married	5805(62.12)	2083(35.88)	3722(64.12)
	Divorced	193(2.07)	78(40.41)	115(59.59)
	Widowed	228(2.44)	77(33.77)	151(66.23)
The main way of medical expenses				
	Self-pay	1864(19.95)	527(28.27)	1337(71.73)
	Resident/employee health insurance	7097(75.95)	2568(36.18)	4529(63.82)
	Commercial Insurance	202(2.16)	82(40.59)	120(59.41)
	Publicly funded	181(1.94)	60(33.15)	121(66.85)
Employment status				
	Employed	2172(23.24)	679(31.26)	1493(68.73)
	Student	4168(44.61)	1559(37.40)	2609(62.60)
	Unemployed	2186(23.39)	701(32.07)	1485(67.93)
	Retired	818(8.76)	298(36.43)	520(63.57)
Smoking status				
	Yes	2069(22.14)	755(36.49)	1314(63.51)
	No	7275(77.86)	2482(34.12)	4793(65.88)
Whether alcohol was consumed in the past year				
	No	5384(57.62)	1783(33.12)	3601(66.88)
	Had drunk in the past month	2823(30.21)	1058(37.48)	1765(62.52)
	Had drunk before the past month	1137(12.17)	396(34.83)	741(65.17)

### Information on participants’ purchase and use of antibiotics for self-medication

**Fig. 2:**
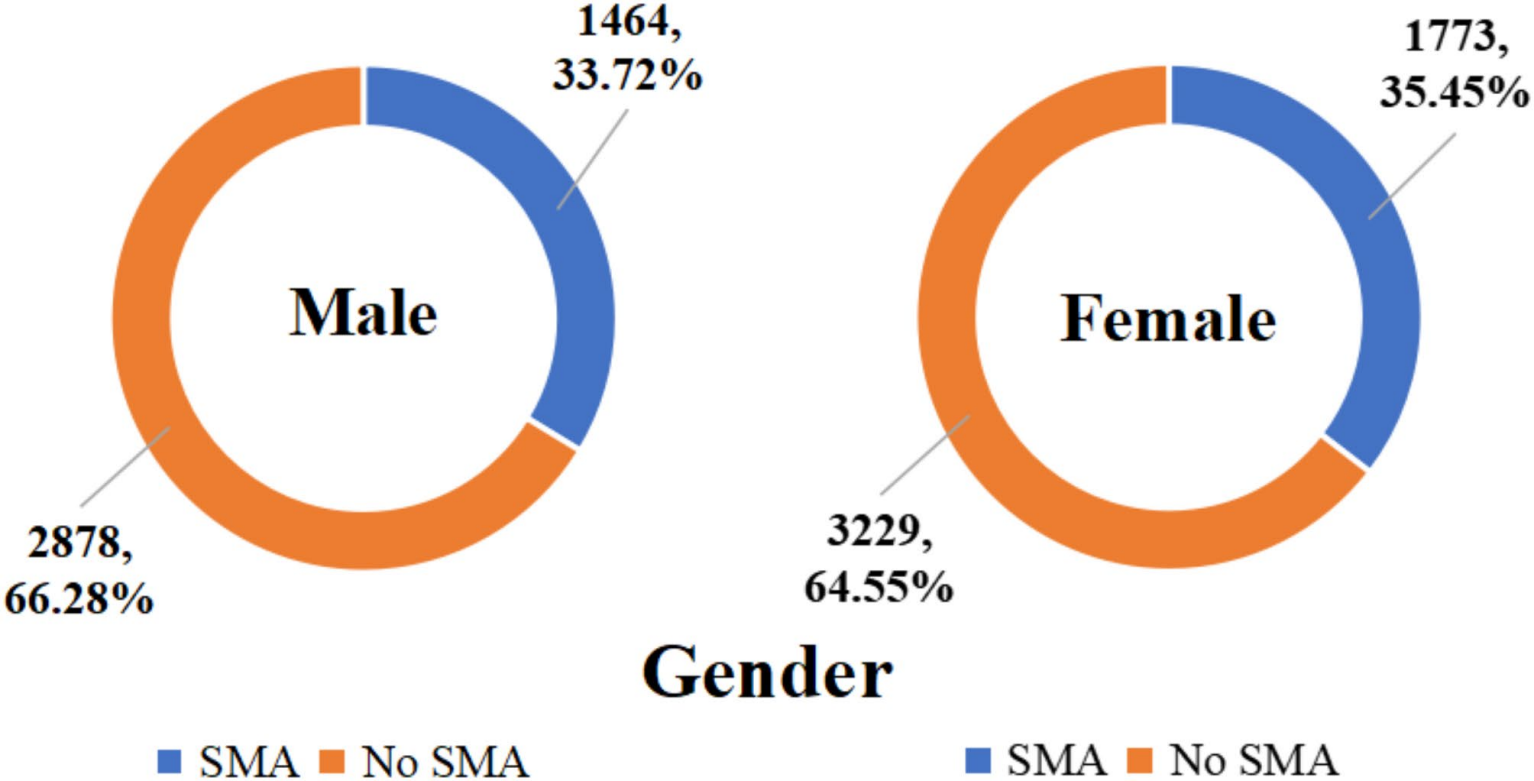
Prevalence of self-medication with antibiotics among Chinese residents by gender groups in 2021

**Fig. 3:**
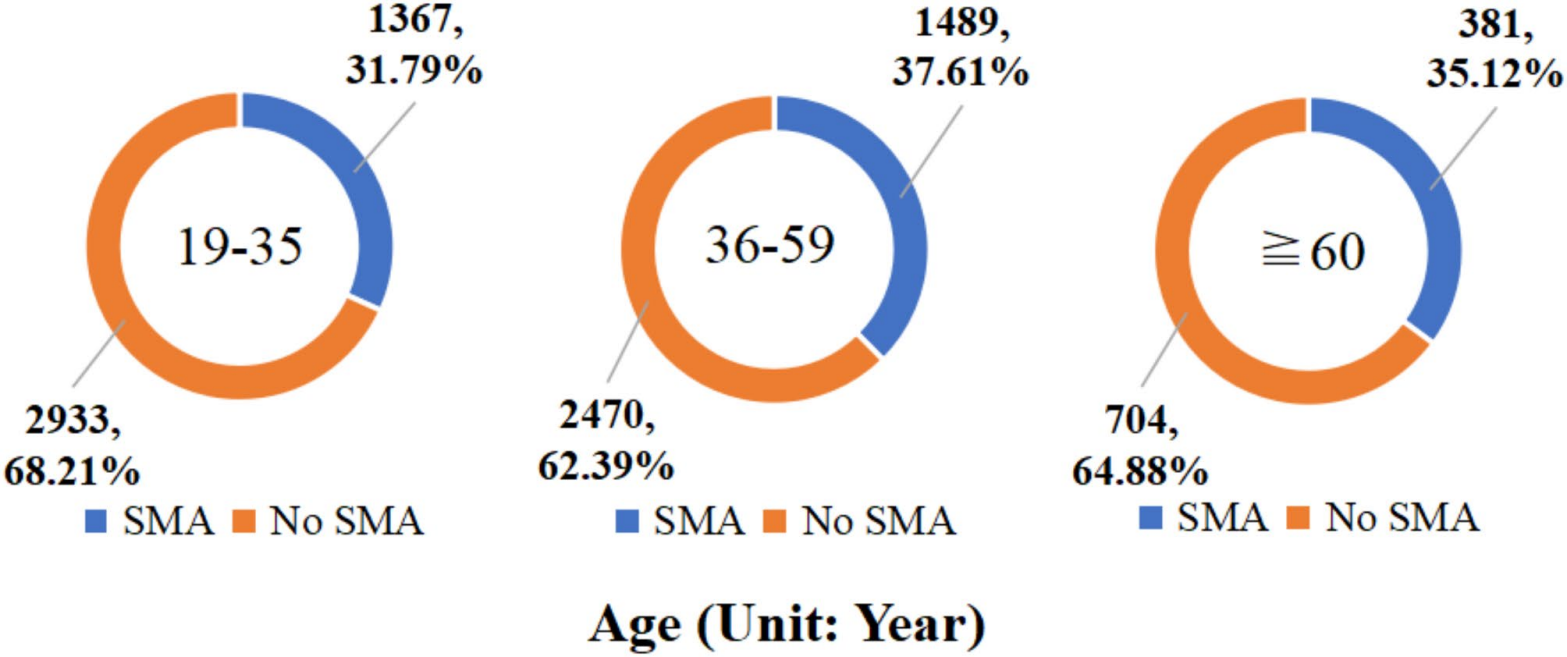
Prevalence of self-medication with antibiotics among Chinese residents by age groups in 2021

**Fig. 4:**
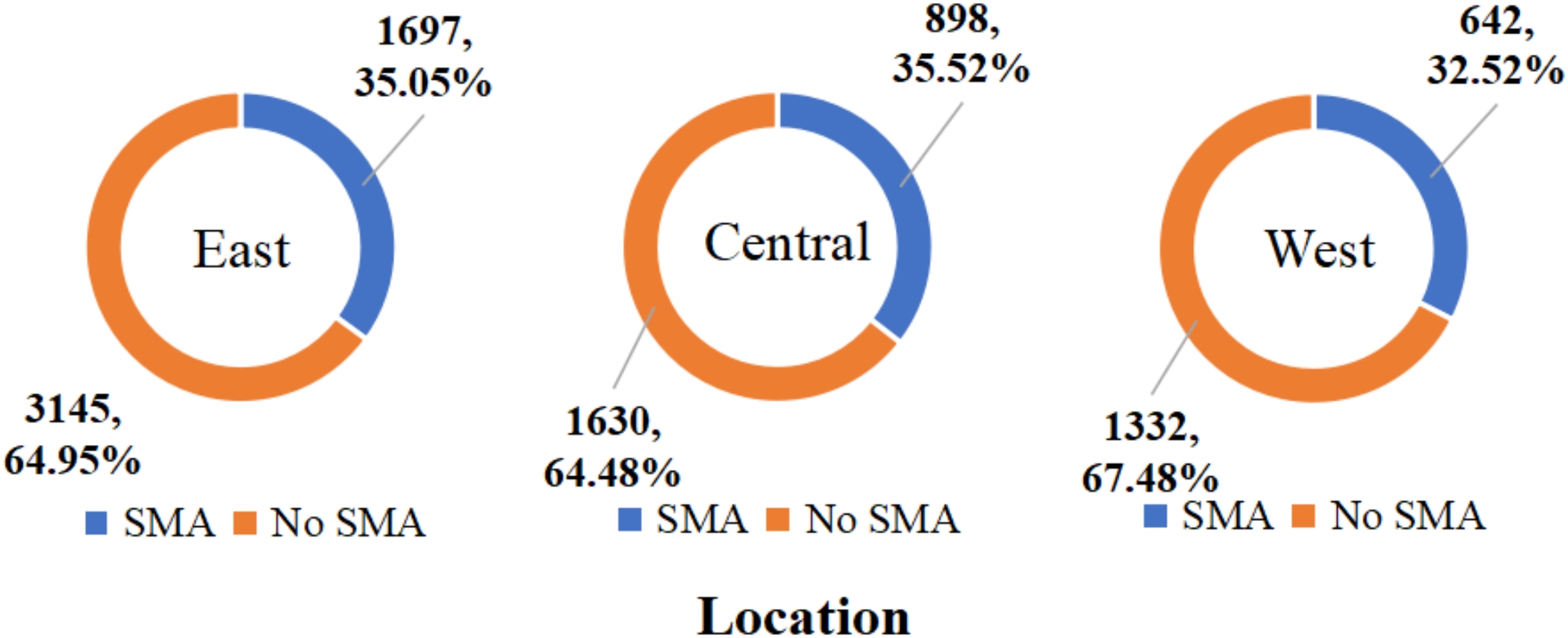
Prevalence of self-medication with antibiotics among Chinese residents by geographic locations in 2021

In this research, we examined the key factors considered by 3,237 participants who engaged in self-medication with antibiotics. Among these respondents, the prevalence of self-medication with antibiotics was 33.72% (1,464/4,342) among males and 35.45% (1,773/5,002) among females. When stratified by age, the prevalence of self-medication with antibiotics was 31.79% (1,367/4,300) among young individuals aged 19–35 years, 37.61% (1,489/3,959) among middle-aged individuals aged 36–59 years, and 35.12% (381/1,085) among older individuals aged 60 years or above. Geographically, the prevalence of self-medication with antibiotics was 35.05% (1,697/4,842) in eastern China, 35.52% (898/2,528) in central China, and 32.52% (642/1,974) in western China. Detailed results can be seen in Figs. 2, 3 and 4.

**Fig. 5:**
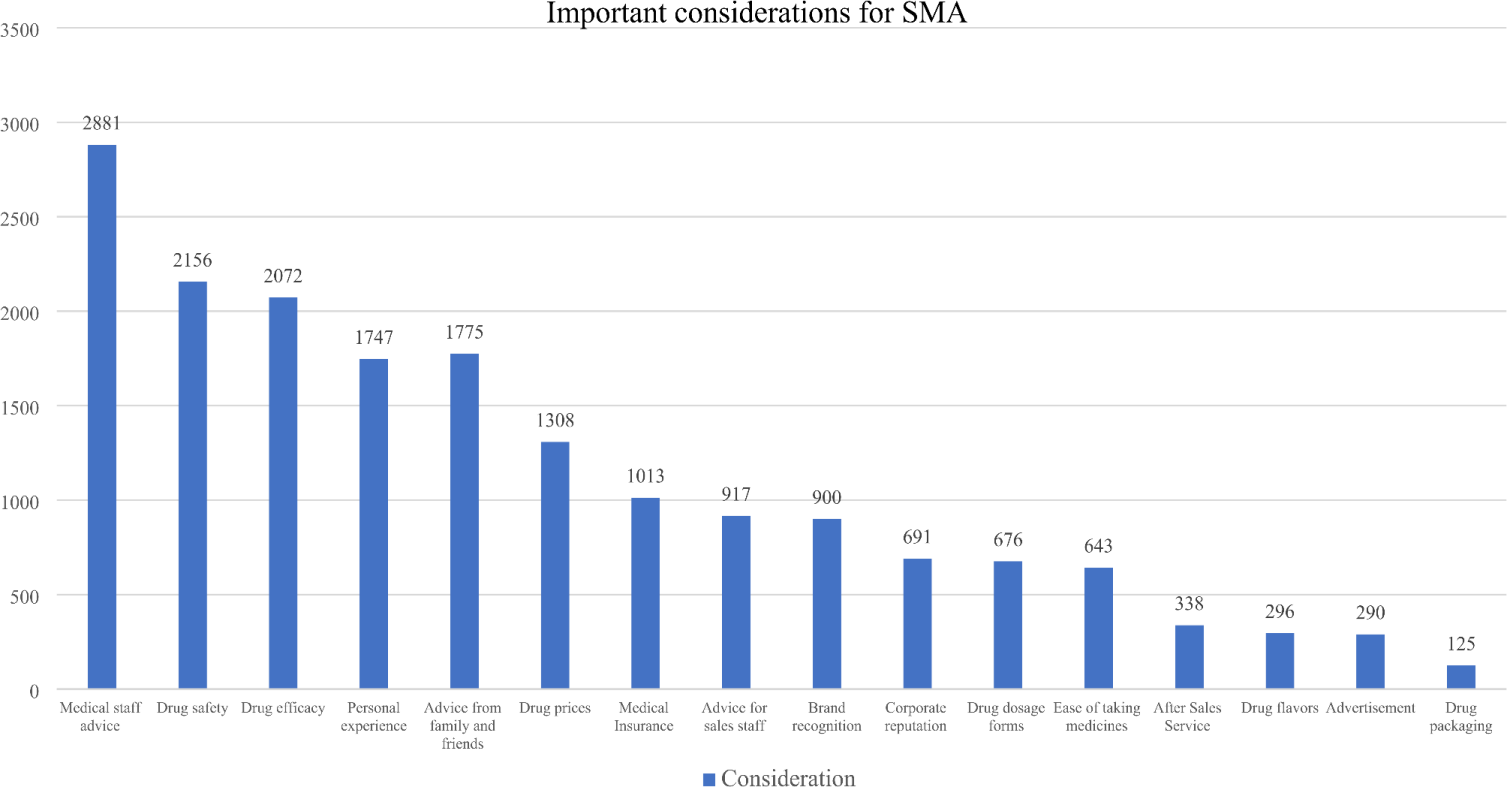
Descriptive statistics of important considerations for participants when purchasing and using antibiotics in 2021 China

The percentage of respondents with SMA who considered physician advice as an important consideration was 77.97% (2,524/3,237) and the percentage of those who considered pharmacist’s advice as an important factor was 58.85% (1,905/3,237). In this study, suggestions from family and friends (1,398/3,237, 43.19%) and medical staff recommendations (877/3,237, 27.09%) were combined with advice from doctors and pharmacists and the medical staff, taking into account how other studies were combined. The proportion of respondents who considered the advice of medical staff (doctor’s advice or pharmacist’s advice) as an important consideration was 89.00% (2,881/3,237) and the proportion of respondents who considered the advice of friends and family (family advice or friend’s advice) as an important consideration was 54.83%. In addition, the survey found that the top factors considered by respondents when purchasing and using antibiotics for self-medication were the safety of the drug (66.60%, 2,156/3,237), drug efficacy (64.01%, 2,072/3,237), personal experience (53.97%, 1,747/3,237) and the price of the drug (40.41%, 1,308/3,237). Detailed information can be found in Table 2; Fig. 5.

### Univariate binary logistic regression analysis of participant SMA and sociodemographic characteristics

A univariate analysis of variance was conducted using binary logistic regression, focusing on the relationship between the demographic and sociological characteristics of the survey respondents and SMA. The results of the univariate logistic regression with whether they had ever purchased and used antibiotics on their own as the dependent variable, and the survey respondents’ demographic and sociological characteristics as the independent variables can be seen in Table 3. The results showed that the differences between the survey respondents’ SMAs based on age, location, permanent residence, education level, marital status, the main way of bearing medical expenses, occupational status, smoking status and alcohol consumption in the last year (*P* < 0.05).

**Table 2:** Results of univariate logistic regression analysis of self-medication with antibiotics (SMA) and participants’ demographic and sociological characteristics in 2021 China

Variables	OR	95%CI lower	95%CI upper
Gender (Control group is male)			
Female	1.08	0.99	1.18
Age (Control group is 19–35)			
36–59	1.29	1.18	1.42
≥ 60	1.16	1.01	1.34
Location (Control group is the Eastern part of China)			
The central part of China	1.02	0.92	1.13
The western part of China	0.89	0.80	1.00
Place of residence (Control group is Urban)			
Rural	1.14	1.03	1.25
Education level (Control group is primary education or no education)			
Secondary education	1.22	1.05	1.43
Tertiary education	1.19	1.03	1.38
Monthly income (RMB) (Control group is ≤ 3000)			
3001–6000	1.08	0.97	1.20
≥ 6000	1.08	0.97	1.21
Marital status(Control group is Not married)			
Married	1.19	1.08	1.30
Divorced	1.44	1.07	1.94
Widowed	1.08	0.81	1.44
The way of paying medical expenses (Control group is Self-pay)			
Resident/employee health insurance	1.44	1.29	1.61
Commercial Insurance	1.73	1.29	2.34
Publicly funded	1.26	0.91	1.74
Employment status (Control group is Student)			
Employed	1.31	1.18	1.47
Unemployed	1.04	0.91	1.18
Retired	1.26	1.06	1.49
Smoke habits (Control group is No)			
Yes	1.11	1.00	1.23
Whether alcohol was consumed in the past year (Control group is No)			
Had drunk in the past month	1.21	1.10	1.33
Had drunk before the past month	1.08	0.94	1.24

### Respondents’ scores on the PSSS, NGSES, FHS-SF, PHQ-9

The scores from the Perceived Social Support Scale, New General Self-Efficacy Scale, the Chinese version of the short-form of the Family Health Scale, and the Depression Screening Inventory are presented in Table 4. As the scores for each scale do not follow a normal distribution, the median and interquartile range are used to assess the concentration and spread of the scores. The survey respondents’ scores on each scale were categorized based on the relevant literature.

There were 4,316 (46.19%) participants with high perceived social support status (higher than 60) and the score cases for the perceived social support moderate support status group (37–60) and low support status group (12–36) were combined in the table and recorded as the low score group (12–60), which were calculated in three categories in the subsequent data. There were 4,104 of the participants (43.92%) who had high self-efficacy (scoring above 30 on the NGSES) and 4,250 participants (56.08%) who had low self-efficacy (scoring from 8 to 30). After converting the family health score, participants had 2,860 (30.60%) in the high family health subgroup (scoring 6–10) and 6486 (69.40%) in the low subgroup (scoring 0–5). The Depression Screening Scale is shown here as a score of 0–4 for no likely depression for 4,250 (45.48%) and a score of 5–27 for depression for 5,094 (54.52%). Those who had depressive symptoms were further divided into three different levels of depression based on their scores, with 34.81% of participants had mild depression (3,253/9,344, with scores between 5 and 9), 10.38% had moderate depression (970/9,344, with scores between 10 and 14), and 9.32% had severe depression (871/9,344, with scores between 15 and 27). In the subsequent calculations, depressive states were calculated using the four categories. The rest of the participants’ scale scores are shown in Table 4.

**Table 3:** Participants’ scores on PSSS, NGSES, FHS-SF, and PHQ-9 in 2021 China

	No. of items	Score range	Low group range	High group range	Kolmogorow-Smironov Z	*P* value from the K-S test	Median	Lower quartile—Upper quartile	High score group *n*(%)	Low score group *n*(%)
**PSSS**	12	12–84	12–36	37–84	0.078	< 0.001	60	49.00–70.00	4316 (46.19)	5028 (53.81)
Family support	4	4–28	4–12	13–28	0.089	< 0.001	20	16.00–24.00	4339 (46.44)	5005 (53.56)
Friend Support	4	4–28	4–12	13–28	0.097	< 0.001	20	16.00–24.00	3867 (41.38)	5495 (58.62)
Other personnel support	4	4–28	4–12	13–28	0.088	< 0.001	20	16.00–24.00	4057 (43.32)	5287 (56.68)
**NGSES**	8	8–40	8–30	31–40	0.134	< 0.001	29	24.00–32.00	4104 (43.92)	4250 (56.08)
**FHS-SF**	10	10–50 0–10 (Assignment)	0–5	6–10	0.082	< 0.001	38	33–43	2860 (30.60)	6484 (69.40)
Family Social and Emotional Health Processes	3	3–15	3–11	12–15	0.158	< 0.001	8	6–9	5993 (64.14)	3351 (35.86)
Family Healthy Lifestyle	2	2–10	2–7	8–10	0.183	< 0.001	10	9–13	6372 (68.19)	2972 (31.81)
Family Health Resources	3	3–15	3–9	10–15	0.105	< 0.001	8	7–10	3987 (42.67)	5357 (57.33)
Family External Social Supports	2	0-100	2–7	8–10	0.190	< 0.001	12	10–14	5524 (59.12)	3820 (40.88)
**PHQ-9**	9	0–27	0–4	5–27	0.137	< 0.001	6.17	1–9	4250 (45.48)	5094 (54.52)

### Rank sum test of participants’ SMA and scale scores

The results of the rank sum test, after grouping the respondents according to whether they had antibiotic self-medication behaviors, revealed statistically significant differences in the distributions of scores for Perceived Social Support, Family, Friends, Other People Support Self-Efficacy, Family Health, Family/Social/Emotional Health Process, Family Healthy Lifestyle Score, family health resources, external family social support, and depressive condition, the differences in score distributions were statistically significant(*P* < 0.001). The results of the rank sum test for participants who had purchased and used antibiotics alone (3,237) and those who had not (6,107) are shown in Table 5.

**Table 4:** Results of Wilcoxon rank sum test of participants’ scores on each scale according to self-medication with antibiotics grouping in 2021 China

Variables	Group (Whether SMA)	Mean Rank	Wilcoxon rank sum test	
			Z	*P*
**PSSS**	No	4497.06	-8.652	**< 0.001**
	YES	5003.49		
Family support	No	4501.28	-8.471	**< 0.001**
	YES	4995.54		
Friend Support	No	4537.38	-6.696	**< 0.001**
	YES	4927.42		
Other support	No	4497.84	-8.641	**< 0.001**
	YES	5002.01		
**FHS-SF**	No	4417.26	-12.587	**< 0.001**
	YES	5154.04		
Family Social and Emotional Health Processes	No	4477.54	-9.788	**< 0.001**
	YES	5040.31		
Family Healthy Lifestyle	No	4514.68	-8.020	**< 0.001**
	YES	4970.25		
Family Health Resources	No	4476.49	-9.714	**< 0.001**
	YES	5042.29		
Family External Social Supports	No	4476.95	-9.885	**< 0.001**
	YES	5041.44		
**PHQ-9**	No	4738.87	-3.519	**< 0.001**
	YES	4547.29		
**NGSES**	No	4556.42	-5.774	**< 0.001**
	YES	4891.48		

### Multivariate binary stepwise logistic regression analysis

A multi-factor binary stepwise logistic regression analysis was performed to examine the relationship between participants purchasing antibiotics alone (dependent variable) and their demographic characteristics, as well as graded scores on each scale (independent variables). The Omnibus test result of the established model is *P* < 0.001, the − 2 log-likelihood value is 11807.949, and the Hosmer-Lemeshow test result is *P* = 0.766 > 0.05, indicating that the model is of good quality.

Multivariate binary stepwise logistic regression showed that gender, age, education, presence of metabolic or digestive disorders, primary mode of health care financing, whether alcohol was consumed in the past year, perceived social support score rating, family health score rating, and depression subgroup were associated with SMA among participants. The results showed that the female group was more likely to develop SMA compared to the male group (OR = 1.158, 95% CI 1.052–1.274, *P* = 0.003). Middle-aged people (36–59 years) were more likely to practice SMA compared to young people [19–35 years (OR = 1.272, 95% CI 1.148–1.409, *P* < 0.001)]. Elderly people (≥ 60 years) were more likely to practice SMA compared to young people [19–35 years (OR = 1.286, 95% CI 1.085–1.523, *P* = 0.004)]. Those with secondary education (OR = 1.254, 95%CI 1.059–1.484, *P* = 0.009) or tertiary education (OR = 1.328, 95%CI 1.117–1.578, *P* = 0.001) were more likely to practice SMA relative to those with primary education or no education. Participants whose primary method of payment for medical expenses was resident/employee health insurance (OR = 1.264, 95% CI 1.125–1.420, *P* < 0.001) or who used commercial insurance (OR = 1.618, 95% CI 1.196–2.191, *P* = 0.002) were more likely to practice SMA than those whose primary method of payment for medical expenses was out-of-pocket. Participants who had consumed alcohol in the last month were more probably to practice SMA relative to those who had not consumed alcohol in the past year (OR = 1.232, 95% CI 1.104–1.374, *P* < 0.001). Participants with a high level of family health score (OR = 1.682, 95% CI 1.494–1.893, *P* < 0.001) were more likely to practice SMA than those with a low level of family health score. Relative to participants without depressive symptoms, participants with mild depression were more likely to practice SMA (OR = 1.102, 95%CI 1.004–1.210, *P* = 0.041) and participants with major depression were less likely to practice SMA (OR = 0.829, 95%CI 0.701–0.980, *P* = 0.028). Participants with a high level of perceived social support score (OR = 1.427, 95% CI 1.061–1.920, *P* = 0.019) were more likely to practice SMA than those with a low level of perceived social support score. See Table 6 for specific details.

**Table 5:** Multi-factor binary stepwise logistic regression results with self-medication with antibiotics as the dependent variable in 2021 China

Variables	SE	OR	95%CI lower	95%CI upper
Gender (Control group is male)				
Female	0.049	1.16	1.05	1.27
Age (Control group is 19–35)				
36–59	0.052	1.27	1.15	1.41
≥ 60	0.086	1.29	1.09	1.52
Location (Control group is the Eastern part of China)				
The central part of China	0.052	1.06	0.96	1.17
The western part of China	0.058	0.90	0.81	1.01
Education level (Control group is primary education or no education)				
Secondary education	0.086	1.25	1.05	1.48
Tertiary education	0.088	1.30	1.10	1.55
The main way of medical expenses (Control group is Self-pay)				
Resident / employee health insurance	0.059	1.26	1.12	1.42
Commercial insurance	0.154	1.61	1.19	2.18
Publicly funded	0.169	1.20	0.86	1.66
Whether alcohol was consumed in the past year (Control group is No)				
Had drunk in the past month	0.054	1.27	1.14	1.41
Had drunk before the past month	0.071	1.13	0.99	1.30
FHS-SF (Control group is low score group)				
High score group	0.057	1.62	1.45	1.81
PHQ-9 (Control group is no depressive symptom group)				
Mild or moderate depression group	0.047	1.11	1.01	1.22
Severe depression group	0.085	0.83	0.70	0.98
PSSS (Control group is low score group)				
Medium score group	0.147	1.28	0.96	1.71
High score group	0.151	1.39	1.04	1.87

### Best subset regression

The variables with significance obtained from the best subset regression results were gender, age, education, The main way of medical expenses, alcohol consumption, family health level, depression level, perceived social support level. Consistent with the results obtained from Multivariate binary stepwise logistic regression analysis. The explanatory and predictive power of the model obtained by the stepwise regression method was improved by performing model robustness tests to ensure the validity of the findings.

**Table 6:** Optimal subset regression model results with self-medication with antibiotics as the dependent variable in 2021 China

Variables	SE	OR	95%CI lower	95%CI upper
Gender (Control group is male)				
Female	0.053	1.20	1.08	1.33
Age (Control group is 19–35)				
36–59	0.053	1.26	1.14	1.40
≥ 60	0.087	1.26	1.06	1.49
Location (Control group is the Eastern part of China)				
The central part of China	0.052	1.06	0.95	1.17
The western part of China	0.058	0.90	0.80	1.01
Education level (Control group is primary education or no education)				
Secondary education	0.086	1.25	1.06	1.48
Tertiary education	0.088	1.33	1.12	1.58
The main way of medical expenses (Control group is Self-pay)				
Resident / employee health insurance	0.060	1.26	1.13	1.42
Commercial insurance	0.154	1.62	1.20	2.19
Publicly funded	0.169	1.21	0.87	1.68
Smoke or not (Control group is No)				
Yes	0.064	1.13	1.00	1.29
Whether alcohol was consumed in the past year (Control group is No)				
Had drunk in the past month	0.056	1.23	1.10	1.37
Had drunk before the past month	0.071	1.12	0.97	1.28
FHS-SF (Control group is low score group)				
High score group	0.060	1.68	1.49	1.89
PHQ-9 (Control group is no depressive symptom group)				
Mild or moderate depression group	0.048	1.10	1.00	1.21
Severe depression group	0.086	0.83	0.70	0.98
PSSS (Control group is low score group)				
Medium score group	0.147	1.29	0.96	1.71
High score group	0.151	1.43	1.06	1.92
NGSES (Control group is low score group)				
High score group	0.052	0.91	0.82	1.01

### Results of subgroup analysis

Binary stepwise logistic regressions were conducted for each subgroup according to gender, age and location, and eight models were developed (see supplementary material for model details).

In terms of gender, the factors associated with SMA for male respondents included age, whether they smoked, and level of family health (*P* < 0.05). Compared to the model developed for men, female respondents had additional factors associated with SMA such as place of usual residence, education level, the main way of medical expenses, whether they had consumed alcohol in the past 12 months, and level of depression (*P* < 0.05). The male model had additional factor about whether they smoked.

In terms of age, factors associated with SMA in the young population (19–35 years) included gender, location, the main way of medical expenses, whether alcohol was consumed in the past twelve months, level of family health, the perceived level of social support, and level of depression (*P* < 0.05). Factors associated with SMA in the middle-aged population (36–59 years) included the marital status, employment status, family health, and the level of depression (*P* < 0.05). Factor associated with SMA in the older age group (≥ 60 years) was family health (*P* < 0.05).

In terms of location, factors associated with SMA in the eastern Chinese population included age, education level, the main way of bearing medical costs, level of family health, and level of depression (*P* < 0.05). Factors associated with SMA in the central region of China included the main way of bearing medical expenses, and the level of family health and depression (*P* < 0.05). Factors associated with SMA in the Western region included monthly income, employment status, whether alcohol has been consumed in the past twelve months, and level of family health. (*P* < 0.05)

## Discussion

### Current situation of self-medication with antibiotics of Chinese residents

This study found that 34.64% of participants engaged in self-medication by purchasing and using antibiotics on their own. A literature review of short-term antibiotic self-medication in Asian countries yielded a prevalence of 50.8% of antibiotic self-medication in Asian populations [6]. A systematic review of the Chinese population estimated that 38% of Chinese residents buy over-the-counter antibiotics from pharmacies [46]. The ratio is lower than that in most developing countries, such as Vietnam (83.3%) [20] and India (58%) [47], but higher than that in some developed countries, such as UK (5%) and France (18%) [14, 48]. Antibiotic self-medication is a common phenomenon occurring worldwide, with a survey of 35 communities from five continents outside of northern Europe and North America showing SMA incidence rates ranging from 19–100% [1]. Attitudes and practices regarding antibiotic use vary significantly between countries due to cultural, economic, and healthcare system differences [49, 50], or some external factors as, for example, prescribing and dispensing of a wide variety of medications that has been affected during the pandemic [51].

The SMA rates shown in this study for the Chinese population are similar to the results of a 2015 cross-sectional study of residents in randomly selected parks in three cities in western, eastern and central China (Xi’an, Changsha and Nanjing). That study showed that almost half of the respondents (45.7%) stated they had used antimicrobials during the last 6 months and 64.4% of them had self-medicated [52], Another survey conducted in 2018, based on China’s largest online survey platform, involved a total of 15,526 adult Chinese residents from three regions in eastern, central and western China, with 37.1% of participants reporting SMA [2].

The percentage of Chinese SMAs obtained in our study was slightly higher compared to the percentage of SMAs on which the sample size was based. There are several possible reasons for this situation: the impact of the COVID-19 outbreak is one of the reasons; COVID-19 reduced the immunity of the population and COVID-19 patients are prone to co-infections with bacterial infections, so the number of Chinese residents with SMA behaviors could therefore increase. In addition to this, the impact of COVID-19 on the country’s economy and healthcare resources has also limited the feasibility of people going to hospitals [53]. People may self-medicate for reasons such as saving time and economic reasons [54].

### Considerations for the Chinese public when purchasing antibiotics for self-administration

In this research, a significant proportion of respondents emphasized the importance of seeking advice from medical professionals when purchasing antibiotics, and the advice of family and friends was also a consideration, followed by the efficacy of the medication. This finding aligns with studies conducted in India [55] and Estonia [56]. The higher number of people considering doctors’ advice than pharmacists’ advice is related to the fact that there are more doctors than pharmacists and that the Chinese population trusts doctors more than pharmacists. On the other hand, pharmacist training is inadequate and there is a shortage of qualified pharmacists in primary care and pharmacies to meet the needs of the growing number of patients [57].

### Analysis of influencing factors on SMA among Chinese individuals

#### Analysis of the impact of individual-level factors on SMA among Chinese individuals

This study comprehensively discusses the factors associated with demographic and sociological characteristics of Chinese residents at the individual level and SMA. The results showed that gender, age, location, level of education, presence of metabolic diseases, presence of digestive diseases, consumption of alcohol, family health status, depression status, and level of appreciated social support were associated with antibiotic self-medication behavior. Participants aged 36–59 are more likely to engage in self-medication with antibiotics (SMA) compared to those aged 19–35 and those over 59. In China probably because most people in this age group are more focused on their careers and families, and the time and money costs of traveling to the hospital are too high, leading them to choose SMA [58]. A study from Portugal also identified age as a potential determinant for self-medication with antibiotics among people in the Algarve region [59]; however, contrary to the findings from the middle-aged group in China, younger individuals (aged 18–34) in Portugal exhibit more prevalent self-medication behavior. This may be attributed to their limited access to health information and a lack of awareness regarding the risks associated with medications, resulting in a higher risk of self-medication. This finding contrasts with our study’s observation that the risk is higher in the 36–59 age group. It also suggests that antibiotic use behaviors across different age groups may be significantly influenced by cultural and societal factors. In a Sudanese survey involving 1,750 adults [60, 61], women were significantly more likely to self-administer antibiotics, which is consistent with our findings, and a UK survey also found that women with better knowledge and attitudes towards antibiotics were more likely to give antibiotics to others without a prescription. However, a Lithuanian study of the public [62] showed that men were more likely to use antibiotics in self-medication. Another study conducted in China [63] also showed that women were less likely to use antibiotics. Some studies have also indicated that the prevalence of SMA is not significantly correlated with gender [64]. Potential explanations for higher SMA rates in females include differences in patient access, and physician-prescribing behaviors. or both [65]. One study indicated that female patients received 57% more antibiotic prescriptions than male patients [66]. Females are therefore also more likely to have leftover antibiotics at home after receiving prescribed treatment, a factor that may increase the risk of SMA [67].

In terms of educational attainment, those with higher levels of education were more likely to practice SMA, with increased levels of education providing no positive impact on the correct use of antibiotics. More educated individuals may be more confident in their ability to use antibiotics appropriately, and conversely, this belief may be more likely to lead to AMR, in line with the findings of the Italian and Sudanese studies [60, 61].

Individuals who had consumed alcohol within the past month tend to have a higher propensity for self-medication with antibiotics compared to those who have not consumed alcohol in the past year. This finding aligns with a study conducted in Spain [68], where unhealthy lifestyles (such as alcohol consumption and smoking habits) in the Spanish adult population were associated with a higher likelihood of self-medication. Similarly, a Serbian [69] study on self-medication showed that respondents who consumed alcoholic beverages had a 1.5 times higher rate of self-medication compared to those who did not. Individuals who engage in alcohol consumption within the past month are more likely to have alcohol abuse. People who consume alcohol are more likely to self-medicate, and they are also more likely to engage in self-medication practices, so this may be the reason why people with drinking behavior are more likely to practice SMA [70].

#### Analysis of the influence of the characteristics of Chinese residents measured by different scales on SMA

This study also examined the relationship between factors such as depression, family health, perceived social support and SMA, with the possibility that the dependent variable of the socio-spiritual dimension may have a more direct impact on behavior. The findings suggest that individuals with higher scores in family health and perceived social support (PSSS) may be more susceptible to SMA. The characteristics measured by the different household health level characteristics, as assessed by the FHS-SF, tend to score higher among urban residents [71]., and people with high levels of family health are more likely to be urban in location. Urban residents with higher levels of family health and better financial status have a higher density of hospitals and pharmacies in urban areas, are more feasible and convenient to purchase antibiotics both online and offline. Consequently, these individuals may be more likely to purchase and use antibiotics compared to people in rural areas. Differences in antibiotic purchases between rural and urban areas may be a potential reason for the apparent association between household health levels and SMA [72, 73]. A study in China investigated that patients with higher household income and good education had better social support [74]. A 2018 study of factors associated with seeking malaria treatment modalities in three poor urban areas of Ghana showed that individuals with high levels of social support were more likely to use self-medication to treat malaria [75]. This finding suggests that positive social support negatively affects health-seeking behavior [76], so it can be hypothesized that individuals with higher levels of social support will have negative health-seeking behavior and thus tend to purchase and use antibiotics alone for self-medication.

In a study investigating depressive states and predisposing factors in Chinese patients with mild obstructive sleep apnea, it was found that depressed patients scored lower than controls on both the total PSSS score and its two subfactors (family support and social support) [77]. Based on this finding, it can be hypothesized that depressed patients tend to have lower levels of social support compared to healthy individuals in the control group. Combined with the inference above that lower levels of social support in depressed patients correspond to more aggressive health care-seeking behavior and a lower likelihood of self-medication, this may account for the lower likelihood of SMA in patients with major depression relative to those without depression. Research has shown that depression is associated with an increased risk of physical illness episodes [78], including widespread bacterial infections [79]. Thus, individuals who suffer from depression may have a higher frequency of antibiotic treatment compared to those who do not, and so may also increase the prevalence of SMA in this population. There are no studies on the influence of depressive factors on the prevalence of SMA, and depression may have an impact on the behavior of antibiotic self-medication in the population from the behavioral aspect (health care seeking behavior, self-medication behavior) or at the level of illness and social support. As for the finding that residents with mild depression and residents with major depression have opposite differences in the likelihood of SMA compared with residents without depression, the details need to be discussed further.

Compared to residents who were not depressed, individuals with mild depression may have higher rates of SMA, but individuals with major depression may have lower rates of SMA. The impact of depression on the prevalence of SMA can be attributed to treatment motivation and behavior. One study mentioned that most patients reported using medications to cope with depressive symptoms and experience elevated moods regardless of their choice of medication [80]. Moreover, people with depressive symptoms may experience nausea, headaches, dizziness, insomnia and decreased resistance [81], and higher prevalence may be accompanied by a greater need for treatment and may also increase the frequency of SMAs. Another study showed that individuals with major depression are less motivated to seek treatment [82], which may explain the lower prevalence of SMA in individuals who are severely depressed. However, there is also a study that suggests that patients with major depression are more prone to engaging in substance abuse behaviors, including SMA, which contradicts the findings of this study [83].

#### Analysis of the factors associated with SMA at the policy level

At the national level, the main way in which health care costs are paid for is also an important factor influencing the SMA of Chinese Individuals. The findings of a study conducted in Iran, which involved 33 pharmacies, support the results of this study [79]. The study revealed that individuals with health insurance are more inclined to engage in self-medication with over-the-counter drugs compared to those without health insurance. The article mentions that cost savings appear to be the primary factor influencing the decision to seek medications without a prescription. It suggests that the rising costs of healthcare play a significant role in driving individuals to resort to self-administering medications as a means of saving money. The study [84] showed that health insurance reimbursement and drug prices were significant factors influencing self-medication behavior. However, there is also a study conducted in developing countries that demonstrated that patients covered by health insurance had a reduced risk of engaging in self-medication with antibiotics [85].

#### Analysis of the effect of factors significant in the subgroup analysis on SMA

Subgroup analyses among different populations differentiated by sex, age, and location showed a significant effect of smoking on SMA in the male population. Saudi study with students showed that smoking consistently predicted the outcome variables in the study (self-medication, SMA, and intention to self-medicate) [86], which the authors suggest may be explained by the fact that the behaviors of smoking, self-medication, and SMA all involve risk-taking and the need to accept uncertain consequences. The fact that students who smoked would still try SMA despite strong evidence of risk may have increased students’ confidence in trying to indulge in risk-taking behaviors, in line with another researcher’s view [87]. Women in urban areas are more likely to practice SMA than women in rural areas, this may be due to the thought that living in an urban area and raising children, and women’s increased knowledge of medications [86], may also affect the increased prevalence of SMA. This finding aligns with the results from Egypt, where living in urban areas is identified as an independent predictor of self-medication practices [88]. This suggests that urban environments may provide better access to medications and healthcare information, thereby facilitating self-medication behaviors among women. Conversely, the findings from Greece present a contrasting perspective [89]. The study indicates that a significant portion of rural adults prefer to use antibiotics without a medical prescription. This observation may provide an alternative explanation for SMA practices, suggesting that rural populations might be engaging in self-medication due to limited access to healthcare services or a lack of awareness regarding the proper use of antibiotics.

The analysis of the younger populations showed that the geographical location was significant in the younger populations compared to the middle-aged and older populations. Young people located in the west were less likely to practice SMA than those located in the east. Some studies have shown that respondents in the western region [90] have lower SMA than respondents in eastern [91] and southern China [3]. Reduction in antibiotic drug use and trust of patients, more health professionals in middle and western China showed positive feedbacks than those in eastern China [92].

The results of the multi-factor binary stepwise logistic regression showed that individuals residing in the western region and having a per capita monthly household income equal to or exceeding 6,000CNY (869.40USD) were less likely to experience SMA compared to those with a per capita monthly household income below 3,000CNY (434.70USD). This is consistent with the previous observation that those who are financially well-off are less likely to self-medicate with antibiotics.

### Suggestions

There is a need for more research on factors associated with antibiotic self-administration, different types such as qualitative, quantitative, observational, cross-sectional, prospective, or retrospective studies, expanding sample sizes or expanding the areas studied, etc., to best address this public health issue. Underdeveloped regions [93], population income, and health insurance reimbursement factors also have an impact on SMAs, and one study found that a national ban on over-the-counter purchases of antibiotics had a very limited impact, with China still reporting over-the-counter purchases and use of antibiotics. The impact of local influences on common practices—such as those involving patients, pharmacies or pharmacists, prescribers, and healthcare systems—has received limited attention in existing studies. The behavior of pharmacies or pharmacists selling antibiotics without a prescription may be attributed to several factors, including economic motives for higher profits [94], a lack of regulatory enforcement, patient demand and expectations, insufficient knowledge about antibiotics among pharmacists [95]. Additionally, during the COVID-19 pandemic, the rise in self-medication and increased demand for over-the-counter antibiotics have influenced pharmacists’ behavior regarding antibiotic sales without prescriptions [95]. Therefore, further national studies should be conducted to identify environmental factors and develop locally tailored strategies. Without such efforts, it is unlikely that the prevalence of self-medication with antibiotics (SMA) will effectively decrease. The study found that people with a high level of education, family health, and social support were more likely to practice SMA. People with a high level of education and family and social support do not necessarily have the appropriate knowledge of antibiotics and proper antibiotic use behavior. As a result, for China, which has a high proportion of self-medication, and a large population, the management of self-medication and public health education on antibiotic use must be strengthened, both online and offline. The most important consideration for individuals when purchasing antibiotics included the advice of medical staff, so doctors and pharmacists should also be more proactive in guiding the correct use of antibiotics. A gradual change in the population’s perception of antibiotic self-administration is a very fundamental part of the process and individuals should be more careful when using them.

### Advantages and limitations of the study

There are several advantages to this study. Firstly, a national survey was conducted, allowing for the collection of comprehensive and representative data from various segments of the population. This increases the generalizability of the findings and enhances the validity of the study. Additionally, this study analyzed residents’ antibiotic self-treatment behaviors in the context of their demographic characteristics, family health, self-efficacy, navigating social support, and depression, expanding the application of frameworks such as family health and navigating social support. Furthermore, the study offers practical implications for improving national knowledge and management of antibiotics. The targeted recommendations derived from the findings can guide future interventions and policies that aim to promote responsible and appropriate antibiotic use.

This study also has some limitations. Firstly, the data are based on a self-reported responses and may be subjected to memory bias. Secondly, the results of the cross-sectional approach to the study can only be used to discuss the factors associated with the dependent variable and do not reflect trends and patterns in events. Thirdly, because the participants in the study were all based in China, they can only represent the situation of individuals in China and cannot be analyzed for other countries. The behavioral characteristics of residents’ SMA and the significant factors considered may change further under the influence of COVID-19, and the data summary as well as the conclusions need to be updated. Fourth, because the data used in this study came from the China Family Health Index-2021 survey, the total data obtained was much larger than the actual sample size used in this study. In addition, because of COVID-19 restrictions, some participants completed the online questionnaire via face-to-face video interviews, and on-site surveys also required the use of smartphones for questionnaire completion. Consequently, older individuals and those with lower educational attainment may have been less likely to participate in the study due to a lack of smartphones or unfamiliarity with their use. As a result, study participants were generally younger and more educated than the general population, which may influence the study’s findings. In summary, the limitations of the actual implementation of the study with regard to sample collection may have the problem that the sample is not fully representative of the total.

## Conclusion

The prevalence of self-purchased and self-administered antibiotics (SMA) among adult Chinese individuals in this study was 34.64%. The prevalence of SMA among Chinese individuals is lower than that of developing countries such as Malaysia and Pakistan, but higher than that of developing countries such as Saudi Arabia and developed countries such as France and Korea. Significant factors that Chinese individuals consider when purchasing antibiotics for self-medication include the advice of medical professionals, drug safety, and drug efficacy. 19- to 35-year-olds with no education or primary education, no health insurance, low household health scores, low perceived social support scores were less likely to engage in SMA. Female, individuals aged 36–59 and aged 60 years or older, who were highly educated, had a primary form of health insurance in the form of resident/employee health insurance or commercial insurance, had high household health scores and high perceived social support scores, were more likely to engage in SMA. Individuals with mild depression are more likely to engage in SMA than those without depression, and individuals with severe depression are less likely to engage in SMA than those without depression.

The government should conduct further research to explore the relationship between economic and health development and SMA. Based on the findings, antibiotic management policies should be formulated and tailored to local conditions. There is a need to prioritize education on antibiotic knowledge, ensuring that the public is well-informed about the appropriate use of antibiotics. This can be achieved through awareness campaigns and educational initiatives targeting both healthcare professionals and the general population. Improving the training of medical staff on antibiotic use is crucial. Healthcare professionals should be equipped with up-to-date knowledge and guidelines regarding antibiotic prescription and should actively engage in instructing residents on the correct and responsible use of antibiotics. Efforts should also be focused on gradually changing the perception and behavior of Chinese residents regarding antibiotic use. This can be achieved through comprehensive awareness programs that emphasize the importance of rational antibiotic use and the potential risks associated with self-medication with antibiotics.

## Electronic supplementary material

Below is the link to the electronic supplementary material.

Supplementary Material 1

## Data Availability

No datasets were generated or analysed during the current study.
