# Supplementary material for: Prevalence and associated factors influencing the use of antibiotics for self-medication among Chinese residents: a cross-sectional study in 2021
Source: Arch Public Health. 2025 Apr 15;83:107. doi: 10.1186/s13690-025-01579-3 (PMC11998442; doi:10.1186/s13690-025-01579-3)
Supplement: Supplementary file 1 — Supplementary Material 1 [file 13690_2025_1579_MOESM1_ESM.docx]

# Supplementary materials

Table 8 Multi-factor binary stepwise logistic regression results for male participants with whether SMA as an important consideration of the dependent variable

| **Variables** | **SE** | **Wald χ2** | **OR** | **95%CI lower** | **95%CI upper** |
| --- | --- | --- | --- | --- | --- |
| Age (Control group is 19-35） |  |  |  |  |  |
| 36-59 | 0.072 | 8.520 | 1.24 | 1.07 | 1.42 |
| ≥ 60 | 0.107 | 0.176 | 1.05 | 0.85 | 1.29 |
| Smoke or not (Control group is No) |  |  |  |  |  |
| Yes | 0.068 | 7.555 | 1.21 | 1.06 | 1.38 |
| FHS-SF (Control group is Low score group) |  |  |  |  |  |
| High score group | 0.072 | 80.302 | 1.91 | 1.66 | 2.21 |

Table 9 Multi-factor binary stepwise logistic regression results for female participants with whether SMA as an important consideration of the dependent variable

| **Variables** | **SE** | **Wald χ2** | **OR** | **95%CI lower** | **95%CI upper** |
| --- | --- | --- | --- | --- | --- |
| Age (Control group is 19-35） |  |  |  |  |  |
| 36-59 | 0.072 | 12.164 | 1.29 | 1.12 | 1.48 |
| ≥ 60 | 0.126 | 9.530 | 1.48 | 1.15 | 1.89 |
| Place of residence (Control group is Urban) |  |  |  |  |  |
| Rural | 0.072 | 4.553 | 1.17 | 1.01 | 1.34 |
| Education level (Control group is primary education or no education) |  |  |  |  |  |
| Secondary education | 0.118 | 3.481 | 1.25 | 0.99 | 1.57 |
| Tertiary education | 0.124 | 7.997 | 1.42 | 1.11 | 1.81 |
| The main way of medical expenses (Control group is Self-pay) |  |  |  |  |  |
| Resident / employee health insurance | 0.079 | 8.940 | 1.27 | 1.09 | 1.48 |
| Commercial insurance | 0.215 | 7.334 | 1.79 | 1.18 | 2.73 |
| Publicly funded | 0.267 | 0.030 | 0.96 | 0.57 | 1.61 |
| Whether alcohol was consumed in the past year (Control group is No) |  |  |  |  |  |
| Had a drink in the past month | 0.084 | 11.126 | 1.33 | 1.12 | 1.56 |
| Had a drink before the past month | 0.100 | 0.863 | 1.10 | 0.90 | 1.33 |
| FHS-SF (Control group is Low score group) |  |  |  |  |  |
| High score group | 0.071 | 40.624 | 1.57 | 1.37 | 1.81 |
| PHQ-9 (Control group is No Depressive Symptom Group) |  |  |  |  |  |
| Mild or moderate depression group | 0.063 | 3.518 | 1.13 | 1.00 | 1.28 |
| Severe depression group | 0.122 | 3.276 | 0.80 | 0.63 | 1.02 |

Table 10 Multi-factor binary stepwise logistic regression results for young people （19-35） participants with whether SMA as an important consideration of the dependent variable

| **Variables** | **SE** | **Wald χ2** | **OR** | **95%CI lower** | **95%CI upper** |
| --- | --- | --- | --- | --- | --- |
| Gender (Control group is male) |  |  |  |  |  |
| Female | 0.073 | 5.231 | 1.18 | 1.02 | 1.36 |
| Location (Control group is the Eastern part of China) |  |  |  |  |  |
| The central part of China | 0.078 | 0.859 | 1.08 | 0.92 | 1.25 |
| The western part of China | 0.087 | 4.980 | 0.82 | 0.69 | 0.98 |
| The main way of medical expenses (Control group is Self-pay) |  |  |  |  |  |
| Resident / employee health insurance | 0.078 | 11.352 | 1.30 | 1.12 | 1.52 |
| Commercial insurance | 0.211 | 6.434 | 1.71 | 1.13 | 2.58 |
| Publicly funded | 0.218 | 0.389 | 1.15 | 0.75 | 1.76 |
| Whether alcohol was consumed in the past year (Control group is No) |  |  |  |  |  |
| Had a drink in the past month | 0.081 | 10.094 | 1.29 | 1.10 | 1.52 |
| Had a drink before the past month | 0.099 | 1.753 | 1.14 | 0.94 | 1.38 |
| FHS-SF (Control group is Low score group) |  |  |  |  |  |
| High score group | 0.084 | 22.516 | 1.49 | 1.26 | 1.76 |
| PHQ-9 (Control group is No Depressive Symptom Group) |  |  |  |  |  |
| Mild or moderate depression group | 0.072 | 2.586 | 1.12 | 0.98 | 1.29 |
| Severe depression group | 0.116 | 1.585 | 0.86 | 0.69 | 1.09 |
| PSSS (Control group is Low score group) |  |  |  |  |  |
| Medium score group | 0.197 | 0.429 | 1.14 | 0.77 | 1.68 |
| High score group | 0.203 | 2.242 | 1.36 | 0.91 | 2.02 |

Table 11 Multi-factor binary stepwise logistic regression results for middle-aged group （36-59） participants with whether SMAof an important consideration as the dependent variable

| **Variables** | **SE** | **Wald χ2** | **OR** | **95%CI lower** | **95%CI upper** |
| --- | --- | --- | --- | --- | --- |
| Marital status (Control group is Unmarried) |  |  |  |  |  |
| Married | 0.207 | 0.836 | 1.21 | 0.81 | 1.81 |
| Divorce | 0.268 | 7.228 | 2.06 | 1.22 | 3.48 |
| Widowed | 0.407 | 0.011 | 1.04 | 0.47 | 2.32 |
| Employment status (Control group is Employed) |  |  |  |  |  |
| Unemployed | 0.074 | 5.823 | 0.84 | 0.72 | 0.97 |
| Retired | 0.147 | 2.909 | 0.78 | 0.58 | 1.04 |
| FHS-SF (Control group is Low score group) |  |  |  |  |  |
| High score group | 0.080 | 59.819 | 1.85 | 1.58 | 2.17 |
| PHQ-9 (Control group is No Depressive Symptom Group) |  |  |  |  |  |
| Mild or moderate depression group | 0.070 | 2.459 | 1.12 | 0.97 | 1.28 |
| Severe depression group | 0.142 | 6.490 | 0.70 | 0.53 | 0.92 |

Table 12 Multi-factor binary stepwise logistic regression results for older people （≥60） participants with whether SMA as an important consideration of the dependent variable

| **Variables** | **SE** | **Wald χ2** | **OR** | **95%CI lower** | **95%CI upper** |
| --- | --- | --- | --- | --- | --- |
| FHS-SF (Control group is Low score group) |  |  |  |  |  |
| High score group | 0.147 | 14.075 | 1.74 | 1.30 | 2.32 |

Table 13 Multi-factor binary stepwise logistic regression results for eastern population participants with whether SMA is an important consideration of the dependent variable

| **Variables** | **SE** | **Wald χ2** | **OR** | **95%CI lower** | **95%CI upper** |
| --- | --- | --- | --- | --- | --- |
| Age (Control group is 19-35） |  |  |  |  |  |
| 36-59 | 0.071 | 13.232 | 1.30 | 1.13 | 1.49 |
| ≥ 60 | 0.122 | 3.734 | 1.27 | 1.00 | 1.61 |
| Education level (Control group is Primary Education or no education) |  |  |  |  |  |
| Secondary education | 0.130 | 5.816 | 1.37 | 1.06 | 1.77 |
| Tertiary education | 0.133 | 9.945 | 1.52 | 1.17 | 1.97 |
| The main way of medical expenses (Control group is Self-pay) |  |  |  |  |  |
| Resident / employee health insurance | 0.084 | 7.059 | 1.25 | 1.06 | 1.48 |
| Commercial insurance | 0.214 | 5.695 | 1.67 | 1.10 | 2.53 |
| Publicly funded | 0.232 | 0.002 | 1.01 | 0.64 | 1.59 |
| FHS-SF (Control group is Low score group) |  |  |  |  |  |
| High score group | 0.071 | 65.569 | 1.78 | 1.55 | 2.05 |
| PHQ-9 (Control group is No Depressive Symptom Group) |  |  |  |  |  |
| Mild or moderate depression group | 0.065 | 7.877 | 1.20 | 1.06 | 1.36 |
| Severe depression group | 0.116 | 1.004 | 0.89 | 0.71 | 1.12 |

Table 14 Multi-factor binary stepwise logistic regression results for central population participants with whether SMA as an important consideration of the dependent variable

| **Variables** | **SE** | **Wald χ2** | **OR** | **95%CI lower** | **95%CI upper** |
| --- | --- | --- | --- | --- | --- |
| The main way of medical expenses (Control group is Self-pay) |  |  |  |  |  |
| Resident / employee health insurance | 0.105 | 7.091 | 1.32 | 1.08 | 1.62 |
| Commercial insurance | 0.288 | 11.092 | 2.61 | 1.48 | 4.58 |
| Publicly funded | 0.299 | 2.069 | 1.54 | 0.86 | 2.77 |
| FHS-SF (Control group is Low score group) |  |  |  |  |  |
| High score group | 0.095 | 28.210 | 1.66 | 1.38 | 2.00 |
| PHQ-9 (Control group is No Depressive Symptom Group) |  |  |  |  |  |
| Mild or moderate depression group | 0.090 | 0.769 | 1.08 | 0.91 | 1.29 |
| Severe depression group | 0.163 | 3.922 | 0.73 | 0.53 | 1.00 |

Table 15 Multi-factor binary stepwise logistic regression results for western population participants with whether SMA as an important consideration of the dependent variable

| **Variables** | **SE** | **Wald χ2** | | | **OR** | **95%CI lower** | | **95%CI upper** | |  |
| --- | --- | --- | --- | --- | --- | --- | --- | --- | --- | --- |
| Monthly income (RMB) (Control group is ≤3000) |  | |  |  | | |  | |  | |
| 3001-6000 | 0.116 | | 0.311 | 1.07 | | | 0.85 | | 1.34 | |
| ≥6000 | 0.136 | | 6.847 | 0.70 | | | 0.54 | | 0.91 | |
| Employment status (Control group is Student) |  | |  |  | | |  | |  | |
| Employed | 0.134 | | 19.070 | 1.80 | | | 1.38 | | 2.34 | |
| Unemployed | 0.144 | | 2.800 | 1.27 | | | 0.96 | | 1.69 | |
| Retired | 0.199 | | 9.684 | 1.86 | | | 1.26 | | 2.75 | |
| Whether alcohol was consumed in the past year (Control group is No) |  | |  |  | | |  | |  | |
| Had a drink in the past month | 0.112 | | 10.245 | 1.43 | | | 1.15 | | 1.78 | |
| Had a drink before the past month | 0.156 | | 5.203 | 1.43 | | | 1.05 | | 1.94 | |
| FHS-SF (Control group is Low score group) |  | |  |  | | |  | |  | |
| High score group | 0.115 | | 26.244 | 1.80 | | | 1.44 | | 2.26 | |
